# Mechanism and Experimental Study on the Recovery of Rare Earth Elements from Neodymium Iron Boron Waste Using the ZnF_2_ Fluorination Method

**DOI:** 10.3390/ma17235807

**Published:** 2024-11-27

**Authors:** Youwei Liu, Yuan Zhong, Xiang Lei, Jinliang Wang

**Affiliations:** 1 School of Metallurgical Engineering, Jiangxi University of Science and Technology, Ganzhou 341000, China; liuyouwei210@163.com (Y.L.); zhongyuan0050@163.com (Y.Z.); 2 Yichun Lithium New Energy Industry Research Institute, Jiangxi University of Science and Technology, Yichun 336000, China; 3 Fengcheng Nonferrous Metals and Advanced Materials Industrial Research Institute, Jiangxi University of Science and Technology, Fengcheng 331100, China; 4 Jiangxi Chunhua Lithium Industry Co., Ltd., Fengcheng 331100, China

**Keywords:** NdFeB scrap, fluorinated reaction, Box–Behnken design, mixed fluorinated rare earths

## Abstract

We conducted a mechanistic and experimental study on zinc fluoride roasting for the recovery of NdFeB waste to address the difficulties faced during this pyrometallurgical recovery process, such as the high dependence on the quality of raw materials, the high energy consumption involved in roasting transformations, and the low added value of mixed rare earth products. Thermodynamic calculations showed the feasibility of fluorinating rare earths in NdFeB waste, and one-factor experiments were performed. The results showed that at a roasting temperature of 850 °C, a reaction time of 90 min, and 100% ZnF_2_ addition, the fluorination rate of rare earths could reach 95.69%. In addition, after analyzing the mesophase composition of a clinker under different roasting temperature conditions, it was found that, when the roasting temperature exceeded 850 °C, the fluorination rate of rare earths was reduced, which was consistent with the thermodynamic results. On this basis, response surface methodology (RSM) was used to carry out experiments to investigate in depth the effects of various factors and their interactions on the fluorination rate of rare earths, which provides a sufficient experimental basis for the recovery of NdFeB waste via fluorination roasting. The results of this study show that ZnF_2_ addition had the greatest influence on the rare earth fluorination reaction, followed by roasting temperature and roasting time. According to the optimization results of the model, the optimal roasting conditions were determined as follows: 119% ZnF_2_ addition at 828 °C, a roasting time of 91 min, and a rare earth element fluorination rate of 97.29%. The purity of the mixed fluorinated rare earths was as high as 98.92% after leaching the roasted clinker with 9 M hydrochloric acid at a leaching temperature of 80 °C, a liquid–solid ratio of 4 mL/g, and a leaching time of 2.5 h. This study will lay the foundation for promoting the application of pyrometallurgical technology in the recycling of NdFeB waste.

## 1. Introduction

NdFeB magnets are a new type of rare earth functional material with excellent comprehensive performance, low cost, and easy processing, which have been widely used in wind power, hybrid vehicles, and hydraulic turbine generators [1,2,3,4]. Cutting and grinding processes must be carried out during the preparation of NdFeB magnets, and the amount of waste generated during these processes amounts to roughly 25%. By 2030, the market demand for NdFeB magnets will reach a minimum of 240 kilotons and a maximum of 644 kilotons. Concurrently, the amount of waste NdFeB magnets supplied for secondary recycling will reach a minimum of 27 kilotons and a maximum of 54 kilotons. It is evident that the recycling of NdFeB magnets has huge economic value [5,6,7,8]. Since NdFeB magnets are important materials and require a relatively high amount of rare earth elements, recovering these elements from NdFeB magnet waste has significant economic and environmental benefits [9].

Pyrometallurgical processes are high volume and quick, and different rare earth products (rare earth alloys or rare earth metals) can be obtained by choosing different processes, leading to broad application prospects [10,11]. The currently reported pyrometallurgical processes for NdFeB recycling mainly include selective chlorination, liquid–metal extraction, and fluoridation separation [12,13,14,15]. The selective chlorination method for recycling used magnets is based on the fact that rare earth elements are more reactive and combine more readily with chloride ions to form chlorides [16,17]. Lorenz and Bertau [18] investigated the treatment of NdFeB magnets with NH_4_Cl, followed by leaching with acetic acid. Under optimal conditions, the yield reached only 84%. The liquid–metal extraction method uses the difference in the affinity of rare earth elements (REs) and iron for other metals to effectively enrich and separate rare earth elements and iron [19]. In particular, the rare earth element, Nd, can form low-melting-point alloys with Ag, Mg, etc. Sun et al. [20] investigated the behavior of Mg in Nd extraction from industrial NdFeB magnet waste. To prevent the oxidation of NdFeB magnets, the optimum extraction temperature should be below 830 °C. Liquid–metal extraction is not suitable for oxidizing waste or sludge material. The presence of rare earth oxides prevents the dissolution of rare earths in molten magnesium. The fluoride separation method utilizes the fact that the rare earth elements in NdFeB waste are more attractive to fluorine than to iron. Sung Gue Heo et al. [21] compared the effectiveness of fluoride and chloride by carrying out an exchange reaction of magnesium halides (fluoride and chloride) with NdFeB waste. The extraction rate of Nd was 98.64% for the fluoride system, at a roasting temperature of 873 K and a holding time of 12 h, and 84.59% for the chloride system, at a roasting temperature of 1073 K and a holding time of 12 h. Although magnesium fluoride has a high extraction rate for rare earths, the higher holding time increases energy consumption. Hu et al. [22] reported a method for the recovery of rare earth elements from scrap NdFeB using an AlF_3_-NaF melt as the fluorinating agent, and the fluorinated rare earths obtained could be subsequently treated with molten salt electrolysis to directly produce Al-Nd-based alloys; however, this method requires fluorination at 900 °C for 3 h, and the recovery rate of rare earths is only 83%.

There are some areas in which current pyrometallurgical processes require urgent improvement, including (1) strict requirements regarding the quality of the waste material (the scrap must have a low content of impurities, and it needs to be tested and sorted to ensure that the content and proportion of the main components meet the requirements for recycling), with most methods applying to NdFeB alloy waste only, and (2) the low purity of the mixed rare earth elements resulting in a product that cannot be easily utilized. The selective fluorination process is considered one of the pyrometallurgical processes able to overcome the above technical difficulties [23]. Its ability to overcome these difficulties can be explained as follows: (1) rare earth elements are easier to combine with fluorine, and therefore, it is not necessary to apply strict requirements regarding the type and quality of the waste utilized during the fluorination reaction process, and (2) most recovered rare earth products require electrolysis to be converted into metals, while the direct production of fluorinated rare earths increases their added value [21,24]. However, limited studies on fluoride roasting for NdFeB scrap recycling result in insufficient theoretical and process parameter support. Therefore, there is an urgent need for an in-depth study of fluoride roasting for NdFeB scrap recycling to promote the application of pyrometallurgical processes in this field.

ZnF_2_ is stable and does not release harmful gasses at high temperatures. We chose ZnF_2_ as the fluorinating agent in the one-factor condition experiment to study the NdFeB fluoridation recovery’s reaction mechanism and experimental rule. Based on the results of our one-factor experiments, a response surface experiment was designed to establish a mathematical model of the fluorination process of rare earths according to the RSM criterion. The roasting process was optimized by analyzing the experimental results. On this basis, a mixed fluorinated rare earth product with a purity of 98.92% was obtained by acid leaching. Due to their high purity, fluorinated rare earths can be roasted at high temperatures to produce rare earth fluorine oxides, which can be dissolved in acid to obtain high-purity rare earth materials for further separation or directly utilized in the production of rare earth alloys or mixed rare earth metals through molten salt electrolysis. The research results will lay the foundation for promoting the application of pyrometallurgical technology in the recycling of NdFeB waste.

## 2. Materials and Methods

### 2.1. Materials

**Figure 1:**
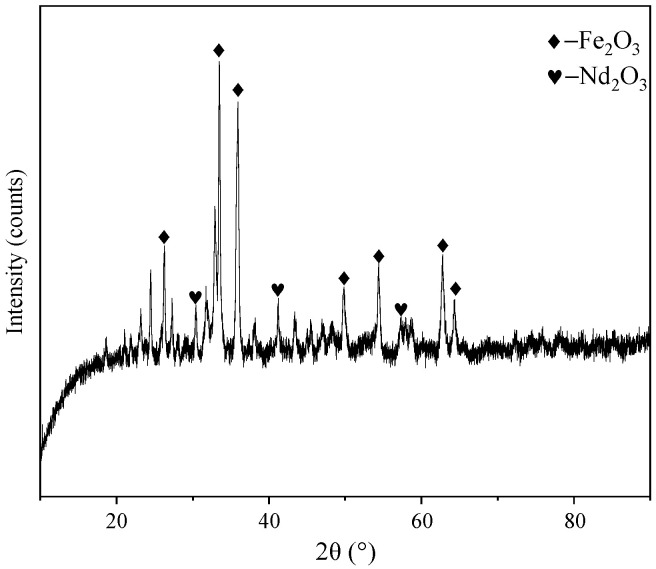
XRD pattern of NdFeB waste.

The raw material used in this study was provided by a NdFeB waste recycling factory in Ganzhou, China, including waste NdFeB magnets and waste materials generated from production. Firstly, the raw material was crushed and roasted to obtain NdFeB oxidized waste. An ICAP-PRO (full-spectrum, direct-reading, inductive coupled plasma emission spectrometer) determined the rare earth content in NdFeB oxidized waste. The proportions of various rare earth oxides are shown in Table 1. The content of rare earth oxides was 26.98%, whereby Nd_2_O_3_, CeO_2_, Pr_6_O_11_, and Gd_2_O_3_ accounted for 94.82% of the rare earth elements. In addition, other impurity elements in the waste were analyzed using an ICAP-PRO analyzer and the chemical titration method, and the results are shown in Table 2. Besides rare earths, there was 48.22% of iron and other trace elements. From the XRD analysis results in Figure 1, it can be seen that the main components of the waste were Fe_2_O_3_ and rare earth oxides.

### 2.2. Procedure

The NdFeB scrap was ground for 6 min using a vibrating mill at 900 rpm to obtain 200-mesh raw materials, which were then mixed with ZnF_2_ (50–150% addition) and subsequently continuously roasted in a muffle furnace at a constant roasting temperature of 750–950 °C for 30–150 min. After roasting, the roasted clinker was ground in a vibrating mill at 900 rpm for 1 min and then placed in a three-neck flask. The roasted clinker was stirred with 5% ammonia at a liquid–solid ratio of 50 mL/g for 60 min at 150 rpm at room temperature and then filtered to remove unreacted ZnF_2_. The filter residue was dried at 80 °C for 12 h and placed in a three-necked flask and acid leached in a constant-temperature water bath at 80 °C for 2.5 h with the addition of 9 M hydrochloric acid at a liquid–solid ratio of 4 (mL/g) with stirring at 150 rpm, then the acid leachate was filtered. The acid leach residue was dried at 80 °C for 12 h. Next, 0.1000 g of the acid leach residue was dissolved in aqua regia and then analyzed for the purity of the mixed fluorinated rare earths using an ICAP-PRO instrument. The leachate was diluted 50 times with 5 % nitric acid and analyzed for rare earth concentration using an ICAP-PRO instrument. The leaching rate of each REE can be calculated by Equation (1); the total rare earth leaching rate can be calculated by Equation (2), and the total rare earth fluorination rate can be calculated by Equation (3).

(1) $$\eta_{i}=\frac{C_{i}\times V}{m_{0}\times \omega_{i}}\times 100\%$$

(2) $$\eta_{i}=\frac{C\times V}{m_{0}\times \omega }\times 100\%$$

(3) χ=1−η

where $m_{0}$ is the mass of the raw material; $\omega_{i}$ (%) is the content of rare earth element “*i*” in the raw material; $C_{i}$ (g/L) is the concentration of rare earth element “*i*” in the leaching solution; and *V* (L) is the volume of the leaching solution. ω(%) is the raw material’s total content of rare earth elements; *C* (g/L) is the total concentration of rare earth elements in the leaching solution.

In this study, the raw materials were roasted in a KSL-1200X muffle furnace manufactured by Hefei Kejing Materials Technology Co. (Hefei, China). The rare earth content in the raw material, the concentration of rare earths in the leach solution, and the content of fluorinated rare earths in the leach residue were measured using an ICAP-PRO instrument from Thermo Fisher Scientific (Waltham, MA, USA). A field-emission scanning electron microscope (SEM), model MLA650F, produced by FEI (Hillsboro, OR, USA), was used to analyze the microscopic morphology of the leaching slag. The physical composition of the raw materials, roasted sand, and acid leaching slag was analyzed using an Empyrean-type X-ray diffractometer manufactured by Panalytical, (Almelo, The Netherlands). The main test specifications and parameters of the instrument were as follows: the target material was Cu-Kα (λ = 0.15406 nm), which was tested in a continuous scanning mode (2θ = 10°–90° in steps of 0.013°). The Gibbs free energies of the chemical reactions were calculated using the HSC 6.0 software developed by Outotec (Helsinki, Finland), and the temperature was set to 0–1000 °C.

### 2.3. RSM Optimization Process Based on Box–Behnken Design

The effects of roasting temperature, roasting time, and ZnF_2_ dosage on the fluorination rate of rare earths were investigated using the RSM method. Three different levels were selected based on the results of the one-factor test. The Box–Behnken design (BBD) was implemented for the three levels of the three factors. Table 3 shows the codes designed and the corresponding actual levels according to the Box–Behnken design model in the Design Expert 13 Trial. Based on the results of the 17 group design experiments, analysis of variance (ANOVA) was performed using RSM, and the quadratic polynomial shown in Equation (4) was obtained by fitting [25].

(4) $$y=a_{0}+\sum_{i=1}^{3}a_{i}x_{i}+\sum_{i=1}^{3}a_{ii}x_{i}^{2}+\sum_{i=1}^{2}\sum_{j=i+1}^{3}a_{ij}x_{i}x_{j}+\varepsilon$$

where *y* is the response value of RSM (rare earth fluorination rate); $a_{0}$ is the intercept term obtained via ANOVA regression using least squares; $a_{i}$ is the first-order linear coefficient; $a_{ii}$ is the square effect; $a_{ij}$ is the quadratic effect between factors; $x_{i}$ and $x_{j}$ are uncorrelated factors; and ε is the random error term caused by inconsistency between the model predicted and actual measured values.

## 3. Results and Discussion

### 3.1. Thermodynamic Calculation

**Figure 2:**
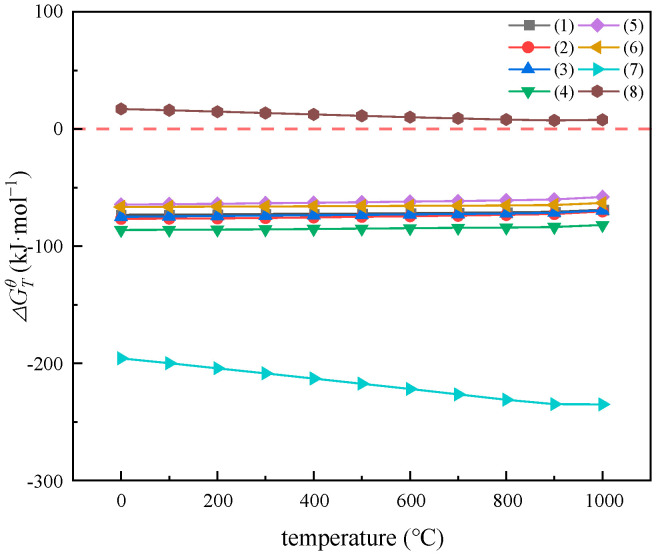
Plot of the reaction ${\Delta G}_{T}^{\theta }$ versus T for the roasting process.

Thermodynamic calculations are essential to analyze the reaction process under study [26]. From the second law of thermodynamics, it is known that, as the absolute value of ${\Delta G}_{T}^{\theta }$ increases, the tendency for the chemical reaction to proceed is greater [27]. Using HSC6.0 thermodynamic software, the positive and negative values of the ${\Delta G}_{T}^{\theta }$ value of each chemical reaction were calculated and compared separately and used as a basis to determine the chemical reaction order and reaction trend. The main chemical reactions of rare earth elements with ZnF_2_ are listed in Table 4, and the calculated ${\Delta G}_{T}^{\theta }$ values are shown in Figure 2.

From Figure 2, it can be seen that the ${\Delta G}_{T}^{\theta }$ of reactions 1 to 6 tends to increase with the increase in reaction temperature, in comparison, that of reaction 7 tends to decrease with the increase in reaction temperature, which indicates that the increase in temperature is not conducive to the fluorination of Nd, Ce, Gd, La, Ho, and Dy but that it is conducive to the fluorination of Pr. For reactions 1 to 7, ${\Delta G}_{T}^{\theta }$ is always negative, indicating that rare earth elements can react with ZnF_2_ to form fluorinated rare earths; whereas for reaction 8, ${\Delta G}_{T}^{\theta }$ is always positive, indicating that Fe_2_O_3_ cannot react with ZnF_2_.

### 3.2. Single-Factor Condition Experiments

#### 3.2.1. Effect of ZnF_2_ Addition on the Fluorination Rate of Rare Earths

**Figure 3:**
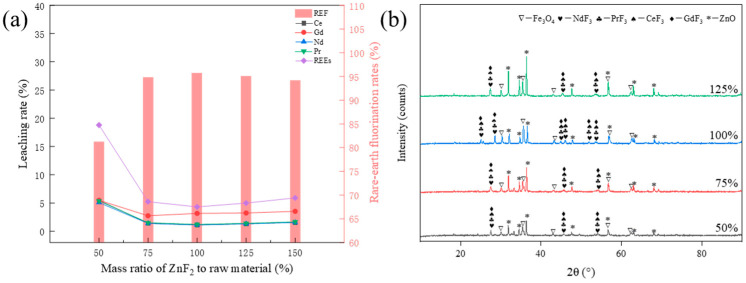
(**a**) Effect of the ZnF_2_-to-raw material mass ratio on rare earth element fluorination rate; (**b**) XRD spectra of the clinker with different ZnF_2_ dosages.

**Figure 4:**
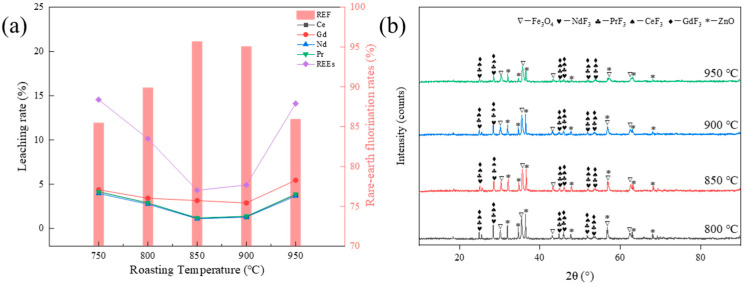
(**a**) Effect of roasting temperature on rare earth element fluorination rate; (**b**) XRD spectra of the clinker with different roasting temperatures.

**Figure 5:**
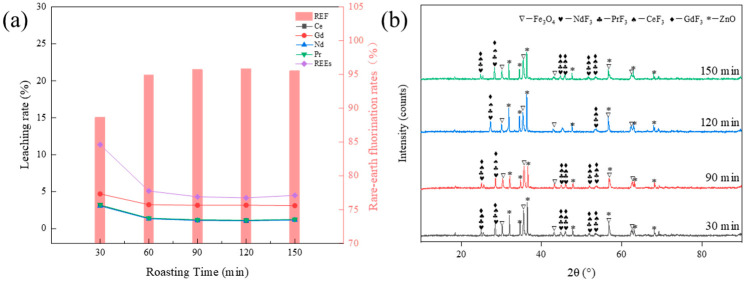
(**a**) Effect of roasting time on leaching; (**b**) XRD spectra of the clinker with different roasting times.

In order to study the effect of different ZnF_2_ additions on the fluorination rate of rare earth elements, the conditions of the roasting process were set as follows: the roasting temperature was 850 °C, and the roasting time was 90 min. Figure 3a illustrates the impact of the leaching rate of rare earth elements at 50–150% of ZnF_2_ addition (in Figure 3a, Figure 4a, and Figure 5a, REF denotes fluorinated rare earth elements, while REEs refers to total rare earth elements). From the curves of the different ZnF_2_ additions shown in Figure 3a, it can be seen that the fluorination rate of rare earths increased from 81.21% to 95.69% when the ZnF_2_ addition increased from 50% to 100%, but the fluorination rate of the rare earths slightly decreased when the ZnF_2_ addition increased to 125%. As shown in Figure 3b, the diffraction peaks of the fluorinated rare earths were gradually enhanced when the addition of ZnF_2_ increased from 50% to 100%, but the diffraction peaks of fluorinated rare earths decreased slightly when the addition of ZnF_2_ increased to 125%. Kinetically, increasing the ZnF_2_ content increases the chance of collision between the elements, which is favorable to the fluorination reaction. Therefore, when the addition of ZnF_2_ increased from 50% to 100%, the fluorination rate also increased. However, when the amount of ZnF_2_ was 125%, the fluorination rate of the rare earths decreased slightly, indicating that the reaction between the two phases is saturated. Moreover, at a constant roasting temperature, excessive ZnF_2_ will make the clinker shrink and harden; the chemical activity will be reduced, and the reaction will be insufficient. Therefore, the fluorination rate of rare earths is optimal when the addition of ZnF_2_ equals 100%.

#### 3.2.2. Effect of Temperature on the Fluorination Rate of Rare Earths

To investigate the effect of the fluorination rate of rare earths at different roasting temperatures, the conditions of the roasting process were set as follows: the addition of ZnF_2_ was 100%, and the roasting time of the material was 90 min. The effect of different roasting temperatures, from 750 to 950 °C, on the fluorination rate of rare earths was investigated, and the results are shown in Figure 4a. As shown in Figure 4a, the rare earth fluorination rate increased significantly from 85.45% to 95.69% as the roasting temperature increased from 750 °C to 850 °C. As the roasting temperature continued to increase, the rare earth fluorination rate decreased. As shown in Figure 4b, the diffraction peaks of NdF_3_, CeF_3_, PrF_3_, and GdF_3_ appeared at the roasting temperature of 800 °C, which means that the oxidized rare earths reacted with ZnF_2_ at this temperature; however, the fluorination rate of rare earths was lower at this time. The diffraction peaks of the fluorinated rare earths were enhanced at a roasting temperature of 850 °C, while the diffraction peaks of the fluorinated rare earths decreased in intensity at 900 °C. From a kinetic point of view, the increase in roasting temperature accelerates the intermolecular collision, thus accelerating the fluorination reaction. Therefore, the rare earth element fluorination rate increases when the roasting temperature increases from 750 °C to 850 °C. However, as the roasting temperature increases, the clinker begins to shrink and harden, and the collision between molecules is weakened, which is unfavorable to the fluoridation reaction, so, when the roasting temperature reaches 900 °C, the fluorination rate of rare earths decreases. Therefore, the optimal roasting temperature was determined to be 850 °C.

#### 3.2.3. Effect of Roasting Time on Fluorination Rate

To investigate the effect of the rare earth element leaching rate at different roasting times, the conditions of the roasting process of the raw material were set as follows: 100% ZnF_2_ addition and 850 °C roasting temperature. The effect of the rare earth elements’ leaching rate at a roasting time of 30–150 min is shown in Figure 5a. From the curve of different roasting times in Figure 5a, it can be seen that the rare earth element fluorination rate increased from 81.21% to 95.69% at a roasting time of 30–90 min, and it tended to be unchanged when the roasting time continued to increase. From the XRD spectra of the clinker with different roasting times in Figure 5b, it can be seen that the peak value of fluorinated rare earths was significantly enhanced when the roasting time increased from 30 to 90 min, and the peak value of fluorinated rare earths essentially remained unchanged when the roasting time continued to be increased. Because the reaction between the oxidized rare earths and ZnF_2_ is a solid-phase reaction, prolonging the roasting time can ensure full contact and reaction between the reactants, so, when the roasting time is increased from 30 min to 90 min, the fluorination rate of rare earths increases. The optimal reaction limit between the reactants is reached by increasing the roasting time, so the rare earth elements’ fluorination rate remains unchanged. Considering the cost and economic benefits, 90 min was chosen as the optimal roasting time.

### 3.3. Box–Behnken Experimental Design

Although RSM cannot explain the response mechanism, the method is able to optimize the test parameters with a minimum number of trials. RSM finds the optimum interval by building a mathematical model and then finds the optimum value in this interval [28]. In RSM, the Box–Behnken design (BBD) method is used to investigate the effect of the interaction of the test factors on the response value [29].

#### 3.3.1. Statistical Analysis and ANOVA

Analysis of variance (ANOVA) is a statistical method used to assess the significance and applicability of regression models [30]. Experiments were conducted based on the BBD principle, and the results are shown in Table 5. Multivariate statistical methods were used to analyze the variance of the fluoridation rate of rare earths, and the results are shown in Table 6. F = 74.60; the signal-to-noise error was only 0.01%, indicating that the constructed regression model is reasonable.

**Figure 6:**
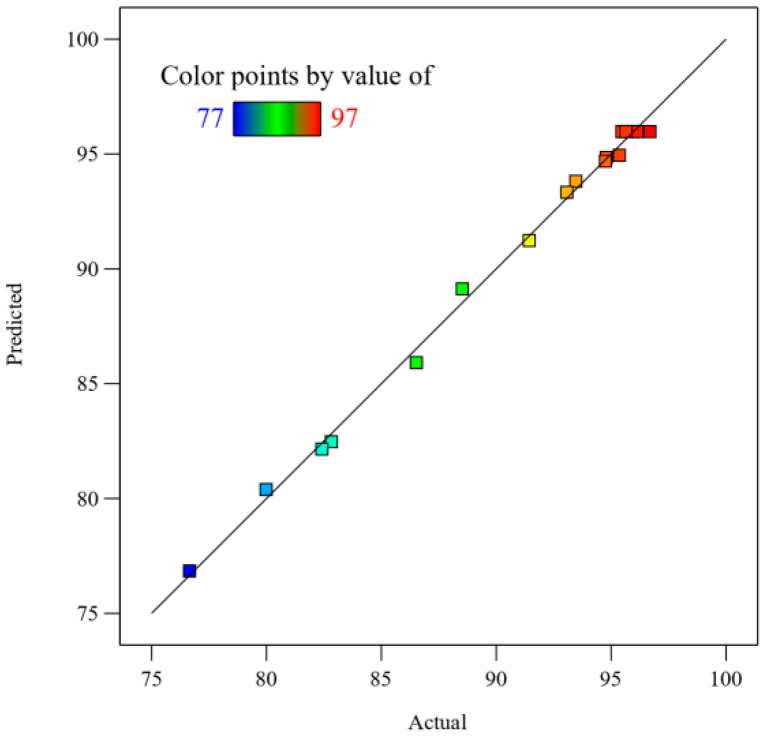
Comparison between predicted and actual values of rare earth element fluorination rates.

First, the 17 trials were counted using multiple linear regression. Then, the quadratic polynomial (5) was obtained using the least squares method. The normal probability plot shown in Figure 6 shows that the predicted values are in very good agreement with the experimental values, indicating that the model is valid. By calculating the correlation between the actual fluoridation rate and the predicted fluoridation rate (R^2^ = 0.9964), it was verified that the model had a good prediction effect. In the process of building the new mathematical model, factors with little influence as determined by the R^2^ value were removed [31]. The corrected regression equation was fitted to obtain a correlation coefficient of $R_{adj}^{2}\;$= 0.9918. In addition, the C.V. was 0.6503%, indicating that the results were reliable. In conclusion, the regression model of the rare earth fluoridation rate established in this paper has a good fitting effect and provides a basis for optimizing the roasting process. The results showed that roasting temperature (A), roasting time (B), and ZnF_2_ dosage (C) were the key factors affecting the fluorination rate of rare earths with a confidence level of 95%. Based on this, the interactions between roasting temperature and roasting time, and roasting temperature and ZnF_2_ dosage were found to be significant. In addition, A^2^ and B^2^ were found to be significant secondary terms.

(5) Rare earth fluorination rate (%) = 95.98 − 0.763A −
0.8435B + 6.43C − 3.62AB + 2.05AC + 0.0369BC − 3.22A^2^ − 1.61B^2^
− 6.66C^2^

#### 3.3.2. Response Surface Optimization Analysis

**Figure 7:**
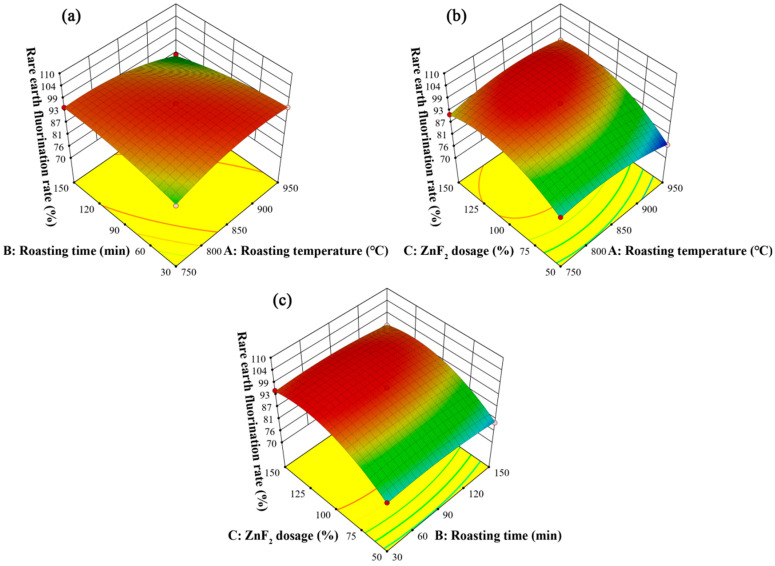
Surface plot of the response of the interaction between different factors on the rare earth element fluorination rate: (**a**) roasting temperature and roasting time, (**b**) roasting temperature and mass ratio of ZnF_2_ to raw material, and (**c**) mass ratio of ZnF_2_ to raw material and roasting time.

The interaction between roasting temperature and roasting time is shown in Figure 7a. When the roasting time was short, the fluorination rate of rare earths increased with an increase in the roasting temperature and then tended to stabilize; however, when the roasting time was long, the fluorination rate of rare earths gradually decreased with the increase in roasting temperature. Similarly, when the roasting temperature was low, the fluorination rate of rare earth elements first increased with the increase in roasting time and then stabilized. However, when the roasting temperature was high, the fluorination rate of rare earths gradually decreased with the increase in roasting temperature. Comparing the effects of the two factors, it can be seen that the effects of roasting temperature and roasting time on the fluorination rate of rare earths are the same. In addition, the trend in the rare earth fluorination rate showed a closer relationship between roasting temperature and roasting time. At the appropriate roasting temperature and roasting time, the reaction system will obtain enough thermal energy to accelerate the intermolecular collision, thus promoting the reaction. In contrast, under the condition of higher roasting temperature or longer roasting time, the clinker will contract and harden, thus hindering the diffusion movement between molecules and lowering the rate of fluorination of rare earths. Figure 7b shows a three-dimensional plot of the effect of roasting temperature and ZnF_2_ addition on the rare earth fluorination rate. In the range of 750~950 °C, the fluorination rate of rare earths always increased and then decreased slightly with the increase in ZnF_2_ addition, which shows that the ZnF_2_ addition greatly influenced the fluorination rate of rare earths. When the amount of ZnF_2_ added is certain, the fluorination rate of rare earths increases and then decreases with the increase in roasting temperature. The fluorination rate of rare earths is relatively high when the roasting temperature is in the middle of the experimental range. As shown in Figure 7c, the interaction between ZnF_2_ dosage and roasting time is not obvious. The effect of increasing the roasting time on the fluorination rate of rare earths was basically unchanged when the amount of ZnF_2_ added was fixed. However, when the roasting time was fixed, the fluorination rate of rare earths increased rapidly and then decreased slightly with the increase in ZnF_2_ dosage. Therefore, the effect of the roasting time on the rare earth fluorination rate was small compared to that of the ZnF_2_ dosage.

Figure 7 shows that, among the three factors of roasting temperature, roasting time, and ZnF_2_ addition, the ZnF_2_ addition had the most significant effect on the fluorination rate of rare earths, and the fluorination rate of rare earths showed a tendency to rise first and then decrease slightly with the increase in ZnF_2_ addition. The experimental results were the same as the law of the one-factor test. This is because, under high-temperature conditions, the rare earths in NdFeB waste can react with ZnF_2_ to obtain insoluble fluorinated rare earths. However, when an excessive amount of ZnF_2_ is added, sintering occurs, leading to the densification of the clinker structure, slowing down the intermolecular collision effect, which is not conducive to the reaction between ZnF_2_ and the rare earth elements, thus reducing the rate of rare earth fluorination.

Using the response surface model, an optimized roasting process was obtained to roast the NdFeB waste with 119% ZnF_2_ addition for 91 min at 828 °C, and the rare earth fluorination rate reached 97.29%. The optimized roasting conditions were tested repeatedly with an error of less than 3%.

### 3.4. Compositional Analysis of Acid Leach Products

The content of mixed rare earth fluorides after hydrochloric acid leaching of the clinker obtained according to the above optimal roasting conditions is shown in Table 7. The content of mixed rare earth fluorides was as high as 98.92%, mainly comprising NdF_3_, CeF_4_, PrF_3_, and GdF_3_, and the content of NdF_3_ was 50.26%; the content of CeF_4_ was 30.97%; the content of PrF_3_ was 10.28%; and the content of GdF_3_ was 3.04%. The content of these four fluorinated rare earths accounted for 94.55% of the total amount of mixed fluorinated rare earths, which is consistent with the proportion of various rare earth oxides in the raw material, indicating that hydrochloric acid has removed impurities such as iron oxide and zinc oxide from the mixed fluorinated rare earths.

**Figure 8:**
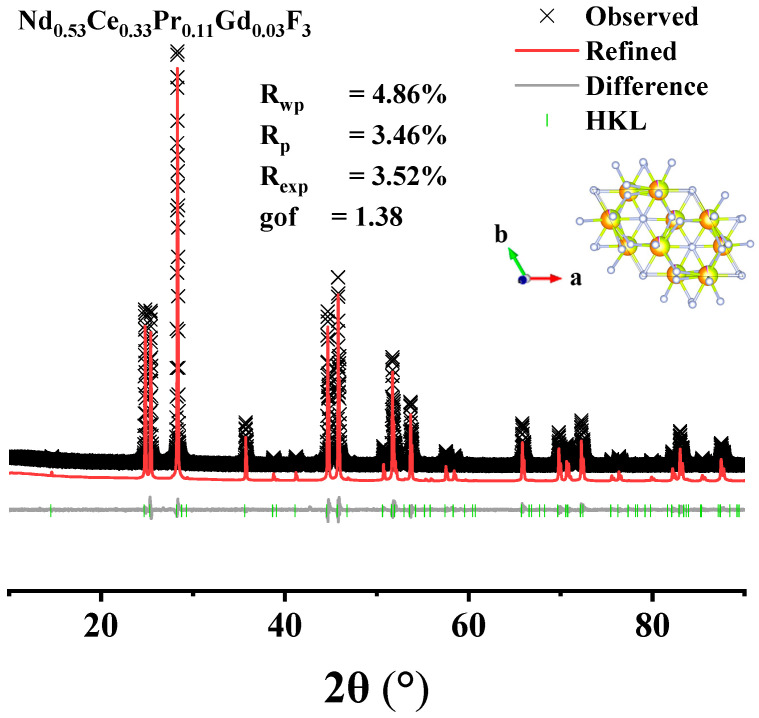
XRD graph of leached products.

**Figure 9:**
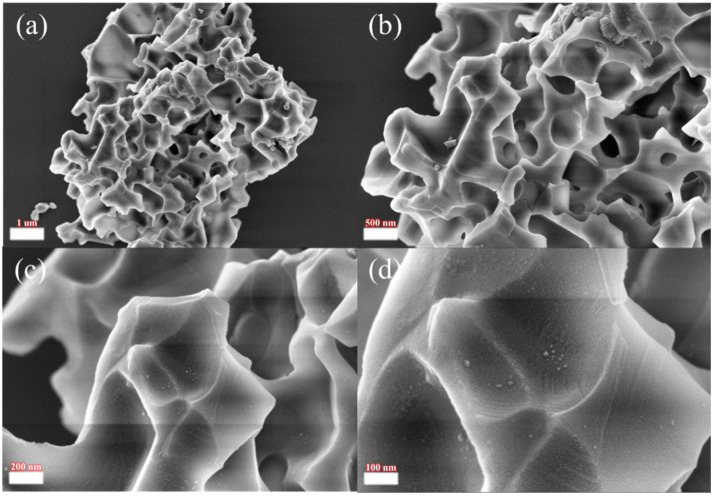
Scanning electron microscope images of the leachate product at different scales: (**a**) 1 µm; (**b**) 500 nm; (**c**) 200 nm; (**d**) 100 nm.

The morphology and structure of the acid-leaching products under the optimal roasting conditions were characterized using XRD and SEM, as shown in Figure 8 and Figure 9. During the Rietveld process, the positions of the atoms were fixed to be the same as that of NdF_3_. The results show that the mixed fluorinated rare earth products existed as doped cells rather than as polymorphs, with the chemical formula of Nd_0.53_Ce_0.33_Pr_0.11_Gd_0.03_F_3_ and the crystal structure of a hexagonal shape. The scanning electron microscope showed that the mixed fluorinated rare earths were irregular and had many holes distributed on the surface, which existed independently. This may be due to the chemical reaction of hydrochloric acid on the material during the acid leaching process, which destroys the impurities such as iron oxide and zinc oxide on the surface and strips them off, leading to the appearance of holes on the surface of the microstructure. Since the main component of the product is fluorinated rare earths and the temperature during the roasting reaction is high, a smooth surface and dense block structure are formed.

### 3.5. Recommendations for the Process

**Figure 10:**
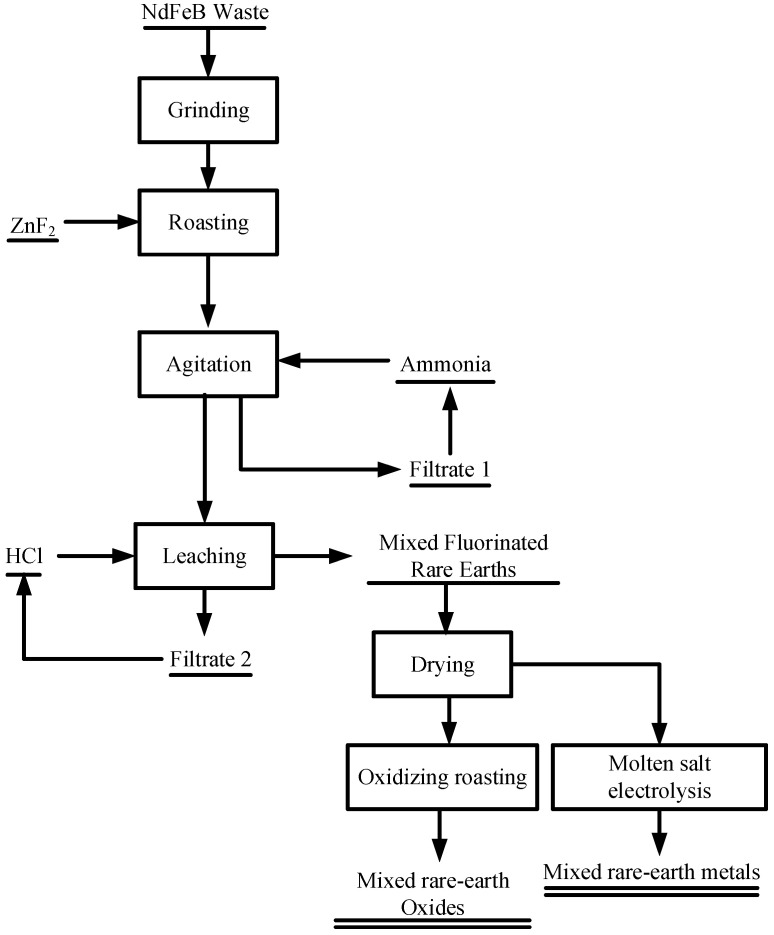
Process flow for recovering rare earths from NdFeB waste through the fluorination reaction.

First, crushed NdFeB scrap is mixed with 119% ZnF_2_ and roasted at 828 °C for 91 min. The roasted clinker is washed with ammonia to remove ZnF_2_, while filtrate 1 is replenished with ammonia and returned to the washing process, thus recycling the ammonia. The washed slag is leached with 9 M hydrochloric acid to obtain fluorinated rare earth slag, while filtrate 2 is supplemented with hydrochloric acid and returned to the acid leaching process, thus realizing hydrochloric acid recycling. Afterward, the fluorinated rare earth slag is dried, oxidized, and roasted to obtain high-purity mixed rare earth oxide products or subjected to molten salt electrolysis to obtain high-purity mixed rare earth metal products. Therefore, the process shown in Figure 10 realizes the synergistic extraction and comprehensive utilization of NdFeB waste, which has good economic benefits and industrial application value.

## 4. Conclusions

This study thoroughly studied the reaction mechanism and process conditions of ZnF_2_ fluoride recovery from NdFeB waste. The research results of this project will lay the foundation for promoting the application of pyrometallurgical technology in NdFeB waste. The main findings and results obtained are as follows:

1. The thermodynamic analysis results show that all the rare earth elements in NdFeB waste can react with ZnF_2_ to produce fluoride. However, increasing the roasting temperature is unfavorable to the fluoride reaction.
2. The optimal roasting process was obtained through one-factor experiments: NdFeB waste was added with 100% ZnF_2_ and then reacted at 850 °C for 90 min, and the recovery rate of rare earths reached 95.69%. In addition, by analyzing the phase composition of the calcined products, the conclusion of the thermodynamic calculations was verified: an excessive calcination temperature is not conducive to the fluorination reaction, which reduces the recovery of rare earths.
3. The BBD model was constructed according to the RSM criterion. It was found that the ZnF_2_ addition had the greatest effect on the rare earth fluorination rate, followed by roasting temperature and roasting time. In addition, the optimal roasting conditions were determined as follows: a roasting temperature of 828 °C, a roasting time of 91 min, and a ZnF_2_ dosage of 119%. The verification experiment demonstrated that the rare earth recovery rate could reach 97.29% under optimal process conditions.
4. Some aspects remain that require further in-depth research and improvement, including the following: (1) detailed experiments on hydrochloric acid purification of fluorinated rare earths and precise control of leaching conditions, which can increase the efficiency of the subsequent fluorinated rare earth purification work; (2) further research on the separation of fluorinated rare earths, which is conducive to improving the value of the product; and (3) optimization of the process conditions and flow to improve the process reliability and practical applicability.

## Figures and Tables

**Table 1:** Proportions of the rare earth oxides in NdFeB waste.

Component	CeO_2_	Pr_6_O_11_	Nd_2_O_3_	Sm_2_O_3_	Gd_2_O_3_	Dy_2_O_3_	Ho_2_O_3_	La_2_O_3_	Eu_2_O_3_	Tb_2_O_3_	Er_2_O_3_	Y_2_O_3_
Content (wt%)	28.82	10.90	51.57	1.91	3.53	2.29	0.79	0.18	<0.2	<0.2	<0.2	<0.2

**Table 2:** Other metal elements contained in NdFeB waste and their contents.

Component	Fe *	Al	Co	K	Ca	Zn	Cu	Ni	Na	Mg	Pb	B
Content (wt%)	48.22	0.96	0.50	0.41	0.27	0.22	0.18	0.15	0.13	0.12	0.12	0.09

* Indicates a chemical titration method.

**Table 3:** Experimental levels and factors design using RSM theory.

Levels	Factors		
	Roasting Temperature (°C)	Roasting Time (min)	ZnF_2_ Dosage (%)
−1	750	30	50
0	850	90	100
1	950	150	150

**Table 4:** The main chemical reactions in the roasting process.

No.	Reactions
1	3ZnF_2_ + Nd_2_O_3_ = 2NdF_3_ + 3ZnO
2	3ZnF_2_ + Ce_2_O_3_ = 2CeF_3_ +3ZnO
3	3ZnF_2_ + Gd_2_O_3_ = 2GdF_3_ + 3ZnO
4	3ZnF_2_ + La_2_O_3_ = 2LaF_3_ + 3ZnO
5	3ZnF_2_ + Ho_2_O_3_ = 2HoF_3_ + 3ZnO
6	3ZnF_2_ + Dy_2_O_3_ = 2DyF_3_ + 3ZnO
7	9ZnF_2_ + Pr_6_O_11_ = 6PrF_3_ + 9ZnO + O_2_ (g)
8	3ZnF_2_ + Fe_2_O_3_ = 2FeF_3_ + 3ZnO

**Table 5:** Box–Behnken design matrix and rare earth fluorination rates.

Run	Roasting Temperature (°C)	Roasting Time (min)	ZnF_2_ Dosage (%)	Rare Earth Element Fluorination Rate (%)
1	950	150	100	86.52
2	850	150	50	79.98
3	950	30	100	94.79
4	850	90	100	95.96
5	750	90	50	82.82
6	850	90	100	95.64
7	850	90	100	96.14
8	850	90	100	95.47
9	750	30	100	88.53
10	750	90	150	91.43
11	950	90	150	93.47
12	850	30	150	95.36
13	850	150	150	93.08
14	850	90	100	96.69
15	750	150	100	94.75
16	850	30	50	82.41
17	950	90	50	76.65

**Table 6:** ANOVA table of the response surface quadratic model.

	Sum of		Mean	F	*p*-Value	
Source	Squares	df	Square	Value	Prob > F	Significant
Model	671.37	9	74.60	215.03	<0.0001	
A—Roasting temperature (°C)	4.66	1	4.66	13.42	0.0080	
B—Roasting time (min)	5.69	1	5.69	16.41	0.0049	
C—Mass ratio of ZnF_2_ to raw material (%)	331.06	1	331.06	954.29	<0.0001	
AB	52.51	1	52.51	151.37	<0.0001	
AC	16.83	1	16.83	48.51	0.0002	
BC	0.0054	1	0.0054	0.0157	0.9039	
A^2^	43.73	1	43.73	126.04	<0.0001	
B^2^	10.92	1	10.92	31.47	0.0008	
C^2^	186.96	1	186.96	538.92	<0.0001	
Residual	2.43	7	0.3469			
Lack of fit	1.53	3	0.5091	2.26	0.2236	Not significant
Pure error	0.9011	4	0.2253			
Cor total	673.80	16				
Fit statistics						
Std. Dev.	0.5890		R^2^		0.9964	
Mean	90.57		Adjusted R^2^		0.9918	
C.V.	0.6503		Predicted R^2^		0.9616	
PRESS	25.85		Adeq Precision		42.3543	

C.V., coefficient of variation.

**Table 7:** Mixed rare earth fluoride content.

REF	CeF_4_	PrF_3_	NdF_3_	SmF_3_	GdF_3_	DyF_3_	HoF_3_	LaF_3_	EuF_3_	TbF_3_	ErF_3_	YF_3_	Total
Content (wt%)	30.97	10.28	50.26	1.74	3.04	1.70	0.54	0.18	0.08	0.10	0.01	0.01	98.92

## Data Availability

The original contributions presented in the study are included in the article, further inquiries can be directed to the corresponding authors.
